# Duration of the preemptive analgesic effects of low‐ and high‐frequency transcutaneous electrical nerve stimulation in rats with acute inflammatory pain

**DOI:** 10.1002/kjm2.12818

**Published:** 2024-03-06

**Authors:** Hideshi Ikemoto, Naoki Adachi, Takayuki Okumo, Oyunchimeg Chuluunbat, Tadashi Hisamitsu, Masataka Sunagawa

**Affiliations:** ^1^ Department of Physiology Showa University Graduate School of Medicine Tokyo Japan

**Keywords:** acute inflammatory pain, opioid receptors, pERK, preemptive analgesic effect, transcutaneous electrical nerve stimulation

## Abstract

Transcutaneous electrical nerve stimulation (TENS) activates various pathways to induce antinociceptive effects, based on the frequencies used. This study evaluates the preemptive analgesic effects and their duration of low‐ (LT: 4 Hz) and high‐frequency TENS (HT: 100 Hz) using a rat model of acute inflammatory pain. Acute inflammation was induced by injecting 1% formalin into the hind paws of rats. LT or HT was applied for 30 min before formalin injection. Pain‐related behaviors, such as licking, flinching, and lifting, were recorded for 60 min postinjection. Immunohistochemistry was used to assess the number of phosphorylated extracellular signal‐regulated kinase (pERK)‐ and c‐fos‐positive cells in the spinal cord. Naloxone, a μ‐opioid receptors (MORs) antagonist, and naltrindole, a δ‐opioid receptors (DORs) antagonist, were administered before TENS application. Pain behavior duration and pERK‐ and c‐fos‐positive cell expression were then measured. LT and HT pretreatment significantly reduced both pain behaviors and the number of pERK‐ and c‐fos‐positive cells postformalin injection. Naloxone and naltrindole partially reversed the effects of LT and HT, respectively. Notably, HT's analgesic effect lasted up to 120 min whereas that of LT persisted for 90 min. LT and HT effectively exerted their preemptive analgesic effects on acute inflammatory pain by inhibiting pERK and c‐fos expression in the spinal cord. HT presented a longer‐lasting effect compared to LT. MOR and DOR activation may contribute to LT and HT's analgesic mechanisms, respectively.

## 1 INTRODUCTION

Acute inflammatory pain from trauma or surgery resolves with wound healing; however, the pain is sometimes prolonged and demonstrates a poor prognosis.^1^ Long‐lasting postoperative pain management is considerably challenging.^2^ Preemptive analgesia is a method for reducing postoperative pain by providing analgesic medication before surgical treatment^2^, ^3^ and includes electrical stimulation therapies, such as electroacupuncture (EA), a combination of acupuncture and pulsed electrical stimulation of pressure points,^4^ and transcutaneous electrical nerve stimulation (TENS).^5^ TENS, in which conductive electrodes are applied to the skin to provide electrical stimulation, has been prominently used for pain relief.^5^, ^6^ This approach has exerted its effects via increasing endogenous opioid secretion,^7^ gamma‐aminobutyric acid secretion,^8^ and descending pain inhibitory system activation.^9^

The transition from acute to chronic pain is partly due to central sensitization in the spinal cord.^2^ Regarding the induction of central sensitization that causes postoperative pain, mechanical stimulation or inflammatory response during surgery activates primary neurons (peripheral sensitization), followed by the secondary neurons in the spinal cord dorsal horn,^2^ which are induced by glutamate receptor N‐methyl‐d‐aspartate receptor activation^10^ and glial cells.^11^ Secondary neuron depolarization induces mitogen‐activated protein kinase pathway activation, including extracellular signal‐regulated kinase (ERK) phosphorylation.^12^, ^13^ Activated ERK (pERK) translocates to the nucleus and activates the cAMP response element‐binding protein (CREB) which is a transcriptional factor involved in long‐term synaptic potentiation and induces immediate‐early gene *c‐fos* expression. These intracellular signalings play a critical role in inducing central sensitization,^2^, ^14^ thus ERK/CREB phosphorylation and c‐fos expression have been used as a marker of pain onset as well as a target for therapeutic strategies.^12^, ^13^, ^14^ Several studies have reported on the efficacy of TENS for pain,^5^, ^6^, ^7^, ^8^, ^9^ but the effects of TENS on central sensitization, ERK activation, and c‐fos expression in the dorsal horn of the spinal cord remain unclear.

The current study investigated the preemptive analgesic effects of low‐ (LT) and high‐frequency TENS (HT) in a rat model of formalin‐induced acute inflammatory pain.^15^ We revealed the involvement of μ‐opioid receptors (MORs) and δ‐opioid receptors (DORs) in the effects of TENS and changes in the ERK and c‐fos activation levels in the dorsal horn. Further, the duration of the preemptive analgesic effects of LT and HT was examined, considering the importance of clarifying the duration of the action of TENS for its clinical application.

## 2 MATERIALS AND METHODS

### 2.1 Animals

Male Wistar rats (6 weeks old, weighing 145–165 g) were purchased from Nippon Bio‐Supp. Center (Tokyo, Japan). Animals were housed in standard plastic cages (for a group habitat, *W* 24 cm × *L* 40 cm × *H* 20 cm) and were kept in our animal facility at 25 ± 2°C and 55% ± 5% humidity, with a light/dark cycle of 12 h during the experiment. Food (CLEA Japan, CE‐2, Tokyo, Japan) and water were provided *ad libitum*. The experiments were performed following the guidelines of the Committee of Animal Care and Welfare of Showa University and the Animal Research: Reporting of In Vivo Experiments (ARRIVE) guidelines for the reporting of animal studies. The Committee of Animal Care and Welfare of Showa University approved all experimental procedures (certificate number: 07026; date of approval: April 1, 2017). All efforts were made to minimize animal suffering and use the minimum number of animals necessary to conduct this study with reliable results. The present study used 154 rats that were randomized and used only once. All observers who scored behavior and performed data analysis were blinded to treatment allocation.

### 2.2 TENS

**FIGURE 1:**
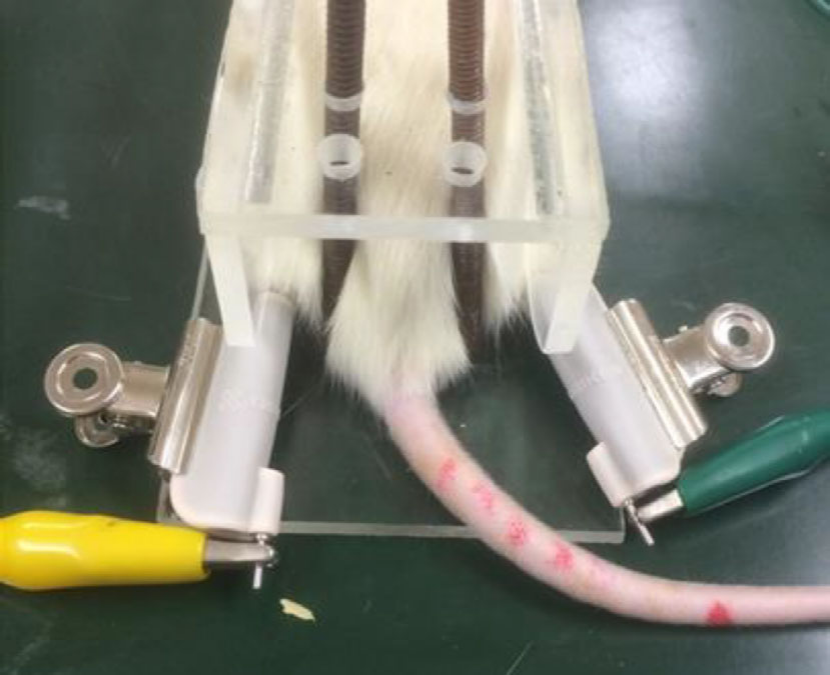
Application of TENS and experimental designs. (A) The rats were fixed in a clear acrylic box (*W* 5 cm × *L* 15 cm × *H* 4 cm) without anesthesia and treated with TENS. The electrodes of the TENS instrument were applied to the bilateral hind paws.

Rats were fixed in a clear acrylic box (*W* 5 cm × *L* 15 cm × *H* 4 cm) without anesthesia and treated with TENS after 7 days of acclimatization. A gel electrode (ELR‐101; OG Wellness, Okayama, Japan) that covered the ankle joints to the tip of the foot on both sides (Figure 1A) was used as the stimulation electrode. This study utilized a SEN‐8203 electric stimulator (Nihon Kohden, Tokyo, Japan), and a square wave stimulus (pulse width: 0.1 ms, stimulus intensity: 1.5 mA, frequency: low‐frequency at 4 Hz or high‐frequency at 100 Hz) was applied for 30 min. This condition was determined based on previous studies.^7^, ^16^ The electrodes were applied without current while being fixed in an acrylic box for the same time in subjects who did not receive TENS.

### 2.3 Acute inflammatory pain model

Acute inflammatory pain was induced via an intraplantar injection of formalin (1%, 50 μL, Polysciences, Warrington, PA, USA) into the right paw using a 30‐G hypodermic needle.^15^ The same volume of saline instead of formalin was administered to rats in the control group. Immediately following the injection, animals were placed into testing cages on a wire‐mesh bottom, and the total time spent engaged in pain‐related behavior was measured for 60 min. Rats were habituated to the cages for 5 days for 60 min before the test. Pain‐related behavior included paw shaking, licking, and lifting from the ground.^15^ The first phase was the duration immediately after formalin administration until 10 min thereafter, whereas the second phase was the duration 10–60 min after formalin administration.^15^

### 2.4 Experimental protocol

We investigated the preemptive analgesic effect of LT and HT using a rat model of formalin‐induced acute inflammatory pain to examine differences in effects depending on endogenous opioid receptor frequency and involvement as a mechanism of action.

**FIGURE 2:**
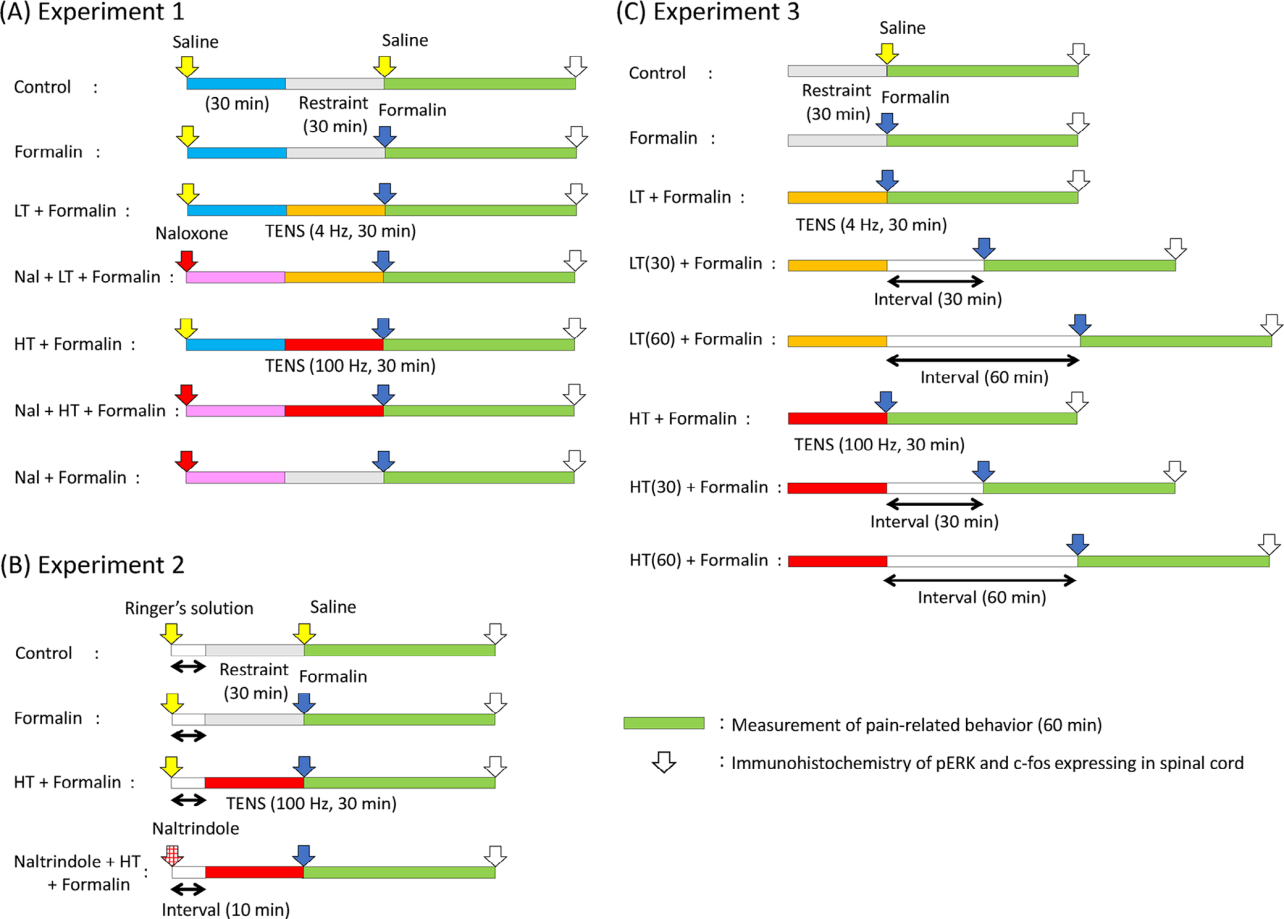
(A) Design of Experiment 1. Rats were categorized into seven groups: control, formalin, LT + formalin (LT‐treated formalin), Nal + LT + formalin (naloxone‐treated LT + formalin), HT + formalin (HT‐treated formalin), Nal + HT + formalin (naloxone‐treated HT + formalin), and Nal + formalin (naloxone‐treated + formalin) groups. TENS was performed at a frequency of 4 or 100 Hz for 30 min immediately before formalin injection. Naloxone was subcutaneously administered 30 min before TENS treatment in the Nal + LT + formalin, Nal + HT + formalin, and Nal + formalin groups. The time spent licking, flinching, or lifting the injected paw was recorded for 60 min after formalin injection, after which the spinal cord (L5) samples were collected for immunohistochemistry. (B) Design of Experiment 2. Rats were categorized into four groups: control, formalin, HT + formalin (HT‐treated formalin), and NTI + HT + formalin (naltrindole‐treated HT + formalin). Naltrindole was intracerebroventricularly administered 10 min before TENS treatment in the NTI + HT + formalin group. (C) Design of Experiment 3. Rats were categorized into eight groups: control, formalin, LT + formalin (formalin treatment immediately after LT), LT(30) + formalin (formalin treatment 30 min after LT), LT(60) + formalin (formalin treatment 60 min after LT), HT + formalin (formalin treatment immediately after HT), HT(30) + formalin (formalin treatment 30 min after HT), and HT(60) + formalin (formalin treatment 60 min after HT). Acute inflammation induction, TENS treatment duration, and formalin test and immunohistochemistry analyses were performed similarly in (B) and (C) experiments, as described earlier. HT, high‐frequency TENS; LT, low‐frequency TENS.

*Experiment 1*: Animals were categorized as follows to investigate the involvement of MORs: a control group (control; *n* = 10), a formalin‐administered group (formalin; *n* = 10), a formalin‐administered group treated with LT (LT + formalin; *n* = 10), an LT + formalin group treated with naloxone before LT (Nal + LT + formalin; *n* = 10), a formalin‐administered group treated with HT (HT + formalin; *n* = 10), an HT + formalin group treated with naloxone before HT (Nal + HT + formalin; *n* = 10), and a formalin group treated with naloxone (Nal + formalin; *n* = 6). Naloxone (3 mg/kg) (N7758; Sigma‐Aldrich, St. Louis, MO, USA), a MOR inhibitor, was subcutaneously administered 30 min before TENS treatment,^17^ whereas rats in the other groups were injected with saline. After LT or HT treatment, pain was induced by formalin administration, after which pain‐related behavior was observed for 60 min (Figure 2A).

*Experiment 2*: Twenty‐four rats were randomly categorized into the following groups to investigate the involvement of DORs in the preemptive analgesic effect of HT: control (*n* = 6), formalin (*n* = 6), HT + formalin (*n* = 6), and HT + formalin treated with naltrindole (NTI) before HT (NTI + HT + Formalin; *n* = 6). Naltrindole (0.1 μg/rat) (N115; Sigma‐Aldrich, St. Louis, MO, USA), a DOR inhibitor, was intracerebroventricularly administered 10 min before HT treatment,^18^ whereas rats in the other groups were injected with Ringer's solution. HT treatment was performed 10 min after administration, after which pain‐related behavior was observed for 60 min (Figure 2B).

*Experiment 3*: We investigated the duration of the analgesic effects of LT and HT treatment on formalin‐induced acute inflammatory pain. The following groups were created: control (*n* = 8), formalin (*n* = 8), LT + formalin (*n* = 8) and HT + formalin (*n* = 8) (formalin treatment immediately after the LT and HT treatment), LT(30) + formalin (*n* = 8) and HT(30) + formalin (*n* = 8) (formalin treatment 30 min after LT and HT treatment), and LT(60) + formalin (*n* = 8) and HT(60) + formalin (*n* = 8) (formalin treatment 60 min after LT and HT treatment). After the injection of formalin, pain‐related behavior was measured for a duration of 60 min following different intervals. Thereafter, rats were intraperitoneally anesthetized with pentobarbital sodium (50 μg/kg; Somnopentyl, Kyoritsu Seiyaku, Tokyo, Japan) and intracardially perfused with phosphate‐buffered saline (PBS) in each experiment. Fifth lumbar spinal cord (L5) samples were harvested after systemic perfusion with 4% paraformaldehyde in 0.1 M of PBS (Figure 2C).

### 2.5 Intracerebroventricular naltrindole administration

The method of intracerebroventricular naltrindole administration was modified from a previous report.^19^ In brief, all rats were deeply anesthetized intraperitoneally with a combination of three anesthetics (medetomidine hydrochloride, midazolam, and butorphanol), and an injection cannula (EIM‐143, Eicom, Kyoto, Japan) was inserted into the lateral ventricle (at the coordinates of AP = −0.8 mm; ML = +1.8 mm; DV = +3.5 mm from the bregma). Naltrindole (0.1 μg/rat) was intracerebroventricularly administered after the recovery period (7 days).^18^ The brain was sampled and the position of the inserted cannula was confirmed after the experiment.

### 2.6 Immunofluorescence

Obtained spinal cord specimens were immersed in 20% sucrose solution for 48 h and subsequently embedded in optimum cutting temperature compound (Tissue‐Tek OCT, Sakura Finetek, Torrance, CA, USA), frozen, and cut into 20‐μm sections using a cryostat (CM3050S, Leica Biosystems, Nussloch, Germany). Sections were incubated overnight at 4°C with rabbit anti‐pERK antibody (1:500, #4370, Cell Signaling Technology, Danvers, MA, USA) and rabbit anti‐c‐fos antibody (1:500, #2250, Cell Signaling Technology). Sections were then incubated for 2 h with fluorophore‐tagged secondary antibody (donkey anti‐rabbit Alexa Fluor 555, 1:1000, #A31572, Thermo Fisher Scientific, Waltham, MA, USA). Nuclei were counterstained with 4′,6‐diamidino‐2‐phenylindole (DAPI, 1:1000, Thermo Fisher Scientific). A confocal laser scanning fluorescence microscope (FV1000D, Olympus, Tokyo, Japan) was used to capture images of the samples. Further, a third person, who is not engaged in the staining process, counted cells, with co‐localization of pERK in the same area of laminae I–II and DAPI, and c‐fos and DAPI in the same area of laminae I–V as pERK‐positive cells and c‐fos‐positive cells, respectively. The mean value was calculated using three sequential sections from each rat.

### 2.7 Statistical analysis

All experimental data were presented as mean ± standard error of the mean. One‐way analysis of variance was used to evaluate the statistical significance of the differences among groups. Tukey's test via Statistical Package for the Social Sciences version 25 (IBM Japan, Tokyo, Japan) was used for post‐hoc comparisons between the groups. All *p*‐values of <0.05 indicated statistical significance. *F*(df1, df2) = *F* represents the result of an analysis of variance, where *F* is the *F*‐statistic, df1 is the degrees of freedom in the numerator (between‐group degrees of freedom), and df2 is the degrees of freedom in the denominator (within‐group degrees of freedom).

## 3 RESULTS

### 3.1 Experiment 1

**FIGURE 3:**
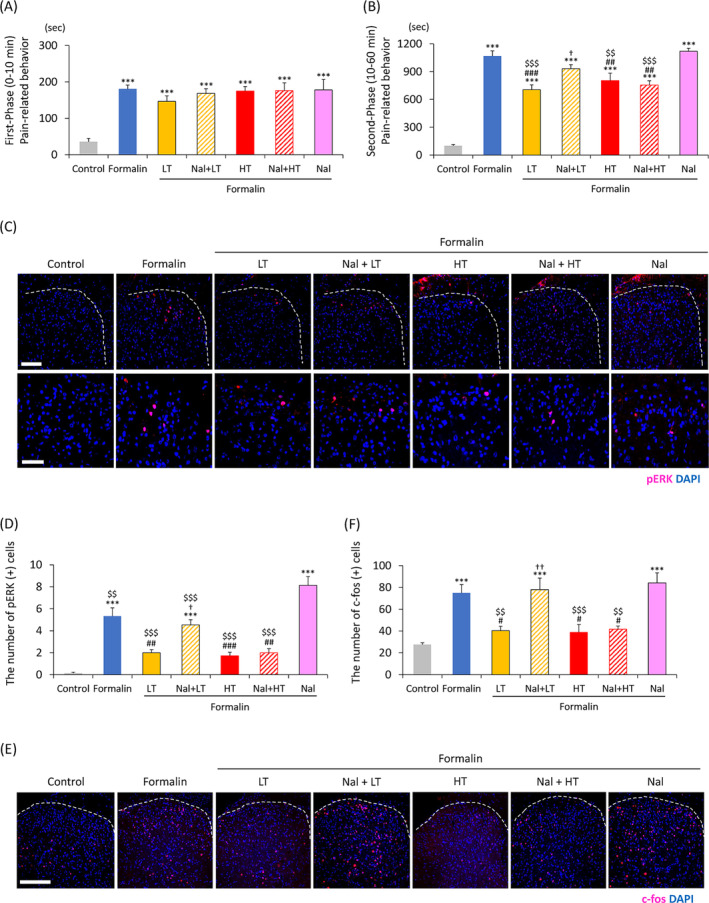
Preemptive analgesic effects of LT and HT with or without naloxone on formalin‐induced acute inflammatory pain. (A and B) Preemptive analgesic effect of LT and HT with or without naloxone on pain‐related behavior in the formalin test, as well as the total duration of pain‐related behavior in the rats during the first (A) and second phases (B). (C–F) Immunohistochemical analysis of pERK and c‐fos in the dorsal horn of the spinal cord. (C) Images of pERK immunoreactivity (red) in the dorsal horn of the spinal cord (L5) (upper; white bar = 100 μm). The lower panels show the enlarged images of the upper panels (lower; white bar = 50 μm). (D) Number of pERK‐positive cells in the right dorsal horn. (E) Images of c‐fos immunoreactivity (red) in the dorsal horn of the spinal cord (L5) (white bar = 200 μm). (F) Number of c‐fos‐positive cells in the right dorsal horn. (A and B), *n* = 10; (C and D), *n* = 5; (E and F), *n* = 6; ***p < 0.001 (vs. the control group); #*p* < 0.05, ##*p* < 0.01, ###*p* < 0.001 (vs. the formalin group); †*p* < 0.05, ††*p* < 0.01 (vs. the LT + formalin group); $$*p* < 0.01, $$$*p* < 0.001 (vs. the Nal + formalin group). HT, high‐frequency TENS; LT, low‐frequency TENS; Nal, Naloxone.

First, we used the effects using naloxone to evaluate the preemptive analgesic effects of LT and HT and the involvement of MORs. Formalin‐induced pain‐related behavior was reported as a biphasic response, similar to the previous study (Figure 3A).^15^ The first phase of the formalin test is caused by the direct chemical activity of nociceptive afferent fibers whereas the second phase is involved in the release of inflammatory mediators.^15^ The formalin group (*F*[6, 59] = 11.9, *p* < 0.001) demonstrated a significantly increased duration of pain‐related behavior in the first phase compared to the control group, but with no significant differences among the five formalin‐treated groups (Figure 3A). The five formalin‐treated groups demonstrated a significantly longer duration of pain‐related behavior in the second phase compared to the control group (*F*[6, 59] = 41.0, *p* < 0.001; Figure 3B). Compared to the formalin group, the LT + formalin group (*F*[6, 59] = 41.0, *p* < 0.001), HT + formalin group (*F*[6, 59] = 41.0, *p* = 0.009), and Nal + HT + formalin group (*F*[6, 59] = 41.0, *p* = 0.001) demonstrated a significantly shorter duration of pain‐related behavior (Figure 3B). Compared to the Nal + formalin group, the LT + formalin group (*F*[6, 59] = 41.0, *p* < 0.001), HT + formalin group (*F*[6, 59] = 41.0, *p* = 0.006), and Nal + HT + formalin group (*F*[6, 59] = 41.0, *p* < 0.001) demonstrated a significantly shorter duration of pain‐related behavior (Figure 3B). Interestingly, naloxone suppressed the effect of TENS only in LT but not in HT (Nal + LT + formalin group, *F*[6, 59] = 41.0, *p* = 0.041; Nal + HT + formalin group, *F*[6, 59] = 41.0, *p* = 0.900; Figure 3B).

The formalin group exhibited more significantly pERK‐positive cells in the dorsal horn of the spinal cord (L5) compared to the control group (*F*[6, 28] = 29.9, *p* < 0.001; Figure 3C,D). This increase was significantly suppressed in the LT + formalin (*F*[6, 28] = 29.9, *p* < 0.001), HT + formalin (*F*[6, 28] = 29.9, *p* < 0.001), and Nal + HT + formalin groups (*F*[6, 28] = 29.9, *p* < 0.001; Figure 3C,D). However, the significant inhibitory effect was not observed in the Nal + LT + formalin group (*F*[6, 28] = 29.9, *p* = 0.009; Figure 3C,D). Additionally, pERK‐positive cells in the Nal + formalin group increased significantly more than all other groups (*F*[6, 28] = 29.9, *p* < 0.001), including the formalin group (*F*[6, 28] = 29.9, *p* = 0.007; Figure 3C,D).

The formalin group demonstrated significantly more c‐fos‐positive cells in the dorsal horn of the spinal cord (L5) compared to the control group (*F*[6, 35] = 11.2, *p* < 0.001; Figure 3E,F). This increase was significantly suppressed in the LT + formalin (*F*[6, 35] = 11.2, *p* = 0.017), HT + formalin (*F*[6, 35] = 11.2, *p* = 0.012), and Nal + HT + formalin groups (*F*[6, 35] = 11.2, *p* = 0.025; Figure 3E,F). However, the significant inhibitory effect was not observed in the Nal + LT + formalin group (*F*[6, 35] = 11.2, *p* = 0.008; Figure 3E,F). The Nal + formalin group exhibited significantly more c‐fos‐positive cells compared to the control group (*F*[6, 35] = 11.2, *p* < 0.001; Figure 3E,F). However, the Nal + formalin group and the formalin group demonstrated no significant difference (*F*[6, 35] = 11.2, *p* = 0.900; Figure 3E,F).

These results indicate that both LT and HT are effective on formalin‐induced acute inflammatory pain by inhibiting pERK and c‐fos expression in the spinal cord dorsal horn and MOR was at least partially involved in the LT's analgesic mechanism.

### 3.2 Experiment 2

**FIGURE 4:**
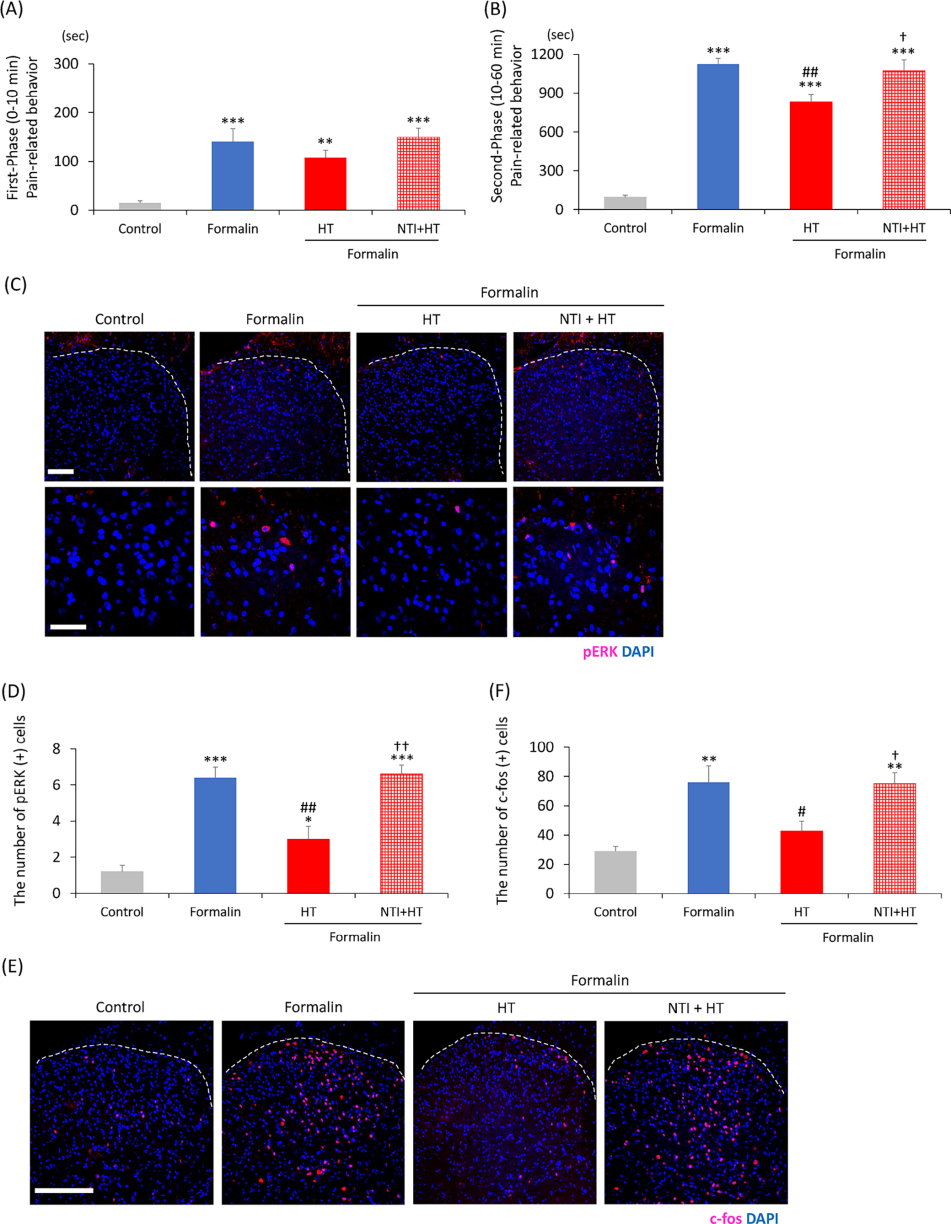
Preemptive analgesic effects of HT with or without naltrindole on formalin‐induced acute inflammatory pain. (A and B) Preemptive analgesic effect of HT with or without naltrindole on pain‐related behavior in the formalin test. The total pain‐related behavior duration in the rats during the first (A) and second phases (B). (C–F) Immunohistochemical analysis of pERK and c‐fos in the dorsal horn of the spinal cord. (C) Images of pERK immunoreactivity (red) in the dorsal horn of the spinal cord (L5) (upper; white bar = 100 μm). The lower panels show the enlarged images of the upper panels (lower; white bar = 50 μm). (D) Number of pERK‐positive cells in the right dorsal horn. (E) Images of c‐fos immunoreactivity (red) in the dorsal horn of the spinal cord (L5) (white bar = 200 μm). (F) Number of c‐fos‐positive cells in the right dorsal horn. (A and B), *n* = 6; (C and D), *n* = 5; (E and F), *n* = 6; *p < 0.05, **p < 0.01, ***p < 0.001 (vs. the control group); #*p* < 0.05, ##*p* < 0.01 (vs. the formalin group); †*p* < 0.05, ††*p* < 0.01 (vs. the HT + formalin group). HT, high‐frequency TENS; NTI, naltrindole.

Naltrindole, a specific antagonist for DORs was tested to investigate the involvement of other types of opioid receptors in the HT's analgesic effect. It has been reported that the analgesic effect of HT on experimentally induced pain is blocked by directly administration of naltrindole into the spinal cord^7^ and the rostral ventromedial medulla.^9^ Therefore, naltrindole was intracerebroventricularly administered to clarify the involvement of DOR distributed in the central nervous system in the preemptive analgesic effect of HT. Pain‐related behavior during the first phase was significantly longer, in the formalin group (*F*[3, 20] = 11.8, *p* < 0.001) than in the control group (Figure 4A), but with no significant differences among the three formalin‐treated groups (Figure 4A). The three formalin‐treated groups demonstrated significantly longer pain‐relative behaviors in the second phase than the control group (*F*[3, 20] = 68.6, *p* < 0.001; Figure 4B). The HT + formalin group (*F*[3, 20] =68.6, *p* = 0.009) demonstrated significantly shorter pain‐relative behaviors than the formalin group (Figure 4B). However, the HT + formalin group exhibited a significant decrease in the duration of pain‐relative behaviors compared to the NTI + HT + formalin group (*F*[3, 20] = 68.6, *p* = 0.0036) (Figure 4B).

The formalin group demonstrated a significantly greater pERK‐positive cell expression compared to the control group (*F*[3, 16] = 25.8, *p* = 0.019; Figure 4C,D). The formalin‐induced increases in pERK‐positive cells were significantly inhibited in the HT + formalin (*F*[3, 16] = 25.8, *p* = 0.006; Figure 4C,D). However, the NTI + HT + formalin group demonstrated no inhibitory effect (*F*[3, 16] = 25.8, *p* = 0.034; Figure 4C,D).

The formalin group exhibited a significantly greater c‐fos‐positive cell expression than the control group (*F*[3, 20] = 10.0, *p* = 0.001; Figure 4E,F). The formalin‐induced increases in c‐fos‐positive cells were significantly inhibited in the HT + formalin group (*F*[3, 20] = 10.0, *p* = 0.028; Figure 4E,F). However, the NTI + HT + formalin group demonstrated no inhibitory effects (*F*[3, 20] = 10.0, *p* = 0.033; Figure 4C,D).

These results indicate that DOR was at least partially involved in the analgesic effect of HT.

### 3.3 Experiment 3

**FIGURE 5:**
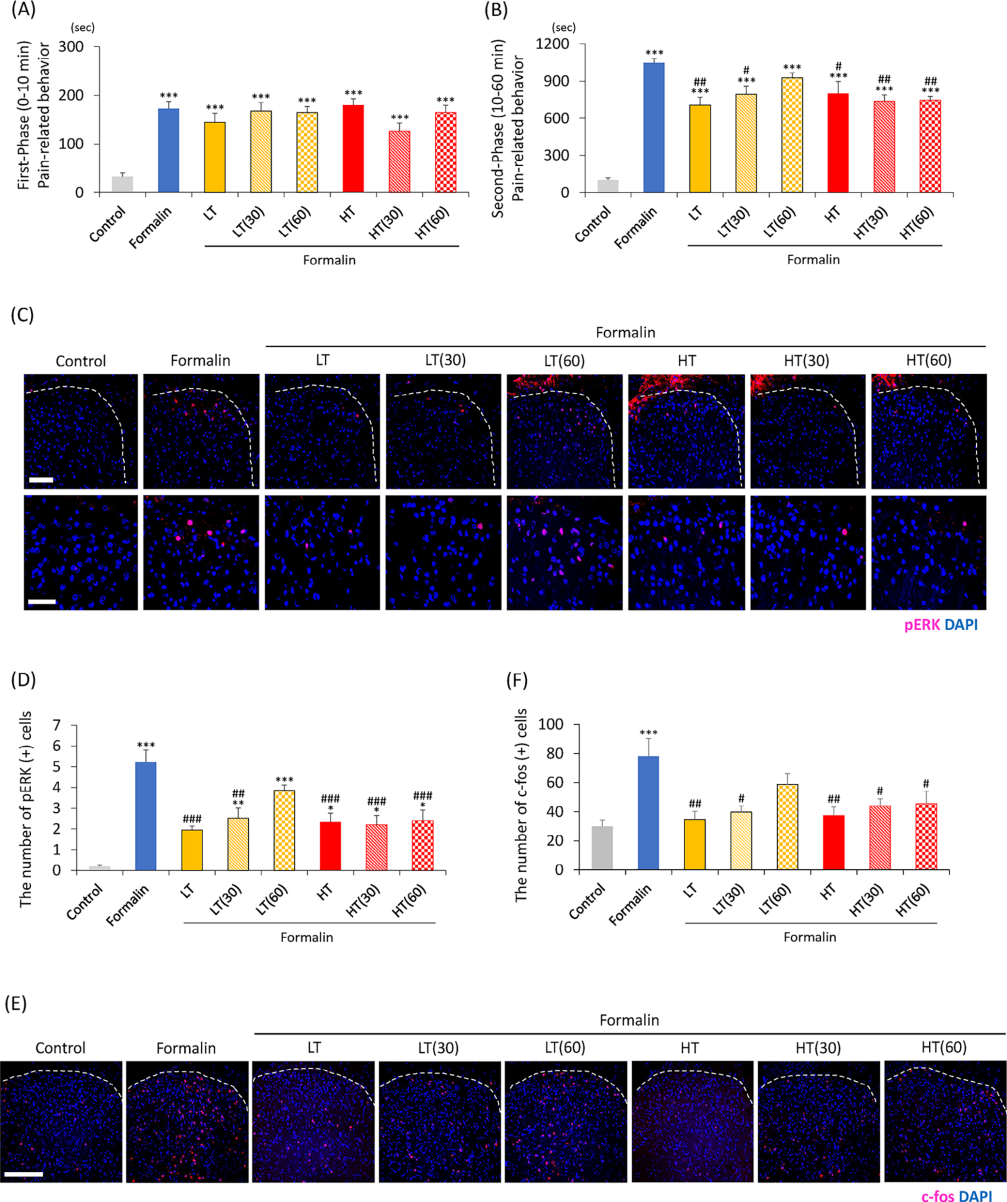
Duration of preemptive analgesic effects induced by LT and HT on pain‐related behavior in the formalin test. The total duration of pain‐related behavior in rats during the first (A) and second phases (B). (C–F) Immunohistochemical analysis of pERK and c‐fos in the dorsal horn of the spinal cord. (C) Images of pERK immunoreactivity (red) in the dorsal horn of the spinal cord (L5) (upper; white bar = 100 μm.). The lower panels show the enlarged images of the upper panels (A, lower; white bar = 50 μm). (D) The number of pERK‐positive cells in the right dorsal horn. (E) Images of c‐fos immunoreactivity (red) in the dorsal horn of the spinal cord (L5) (white bar = 200 μm). (F) Number of c‐fos‐positive cells in the right dorsal horn. (A and B), *n* = 8; (C and D), *n* = 5; (E and F), *n* = 6; *p < 0.05, **p < 0.01, ***p < 0.001 (vs. the control group); #*p* < 0.05, ##*p* < 0.01, ###*p* < 0.001 (vs. the formalin group). HT, high‐frequency TENS; LT, low‐frequency TENS.

We then determined the duration of the action of TENS. Pain‐related behavior during the first phase in the formalin group (*F*[7, 56] = 10.6, *p* < 0.001) was significantly longer compared to that in the control group (Figure 5A); however, no significant differences were found among the seven formalin‐treated groups (Figure 4A). The seven formalin‐treated groups demonstrated significantly longer pain‐relative behaviors during the second phase compared to the control group (*F*[7, 56] = 25.4, *p* < 0.001; Figure 5B). The LT + formalin (*F*[7, 56] = 25.4, *p* < 0.001), LT(30) + formalin (*F*[7, 56] = 25.4, *p* = 0.040), HT + formalin (*F*[7, 56] = 25.4, *p* = 0.048), HT(30) + formalin (*F*[7, 56] = 25.4, *p* = 0.005), and HT(60) + formalin groups (*F*[7, 56] = 25.4, *p* = 0.007) demonstrated significantly shorter pain‐relative behaviors than the formalin group (Figure 5B). However, the LT(60) + formalin group (*F*[7, 56] = 25.4, *p* = 0.755) exhibited no significant difference in pain‐relative behavior duration compared to the formalin group (Figure 5B).

The formalin group demonstrated a significantly greater expression of pERK‐positive cells compared to the control group (*F*[7, 48] = 11.5, *p* < 0.001; Figure 5C,D). The formalin‐induced increases in pERK‐positive cells were significantly inhibited in the LT + formalin (*F*[7, 48] = 11.5, *p* < 0.001), LT(30) + formalin (*F*[7, 48] = 11.5, *p* = 0.001), HT + formalin (*F*[7, 48] = 11.5, *p* < 0.001), HT(30) + formalin (*F*[7, 48] = 11.5, *p* < 0.001), and HT(60) + formalin groups (*F*[7, 48] = 11.5, *p* < 0.001; Figure 5C,D). However, the LT(60) + formalin group demonstrated no inhibitory effect (*F*[7, 48] = 11.5, *p* = 0.337; Figure 5C,D).

The formalin group exhibited a significantly greater c‐fos‐positive cell expression compared to the control group (*F*[7, 40] = 4.8, *p* < 0.001; Figure 5E,F). The LT + formalin (*F*[7, 40] =4.8, *p* = 0.023), LT(30) + formalin (*F*[7, 40] = 4.8, *p* = 0.001), HT + formalin (*F*[7, 40] = 4.8, *p* = 0.005), HT(30) + formalin (*F*[7, 40] = 4.8, *p* = 0.031), and HT(60) + formalin group (*F*[7, 40] = 4.8, *p* = 0.047) demonstrated significantly inhibited formalin‐induced increases in c‐fos‐positive cells (Figure 5E,F). However, the LT(60) + formalin group demonstrated no inhibitory effect (*F*[7, 40] = 4.8, *p* = 0.541; Figure 5E,F).

## 4 DISCUSSION

The formalin‐induced inflammatory pain model demonstrated biphasic pain can be produced by the physical stimulation caused by formalin itself and the inflammatory response.^15^ The injection of formalin into the rodent's paw generates long‐lasting mechanical allodynia and hyperalgesia,^20^, ^21^ which manifest clinically as a result of central sensitization,^22^ thus this model has been recently utilized as not only an acute inflammatory pain model but also a persistent inflammatory pain model.^20^, ^21^ Notably, spinal cord central sensitization in this model has been associated with pain‐related behavior development observed in the second phase.^15^, ^23^ Therefore, inhibiting pain‐related behavior in the second phase is effective in preventing the establishment of central sensitization and the transition from acute pain to chronic pain.^1^, ^20^ Recent studies revealed the involvement of ERK activation in the dorsal horn in synaptic potentiation,^12^, ^23^ which plays a crucial role in central sensitization and the resultant pain hypersensitivity.^23^ Evidence has indicated that pharmacological inhibition of ERK activation in superficial spinal cord neurons reduced pain behaviors.^12^ Furthermore, activated ERK translocates from the cytoplasm to the nucleus, activates CREB, and induces c‐fos expression.^24^ CREB phosphorylation by pERK is required for stable c‐fos expression.^25^ In particular, c‐fos expression proves continuous and stable ERK expression.

The current study revealed that both LT and HT significantly suppressed the dramatic increase in pain‐related behavior duration following formalin administration in the second phase. Consistent with pain‐related behavior duration, LT and HT significantly decreased the increase in the number of pERK‐ and c‐fos‐positive cells caused by formalin administration. However, naloxone administration antagonized these effects of LT. These results indicated that LT‐induced pain relief, ERK inhibition, and c‐fos activation were partially dependent on MOR activation.

Human studies revealed that LT increases plasma β‐endorphin levels, an agonist of MOR.^26^ Further, low‐frequency EA has released endogenous MOR agonists and attenuated the induction of inflammatory pain.^27^ Sluka et al. revealed that naloxone antagonized the analgesic effects of LT on a rat model of carrageenan‐induced arthritis.^7^ Kawasaki et al. revealed that morphine administration suppressed the mechanical and chemical stimulation‐induced increase in pERK levels in the spinal cord dorsal horn.^28^ Furthermore, Liao et al. revealed that the endogenous MOR agonist released after 2‐Hz EA prevented ERK phosphorylation through protein kinase C activity downregulation, which activates ERK in mice with inflammatory pain.^29^ Intrathecal administration of an inhibitor of MEK, which is an upstream protein of ERK, blocked pain‐related behavior in the second phase in the formalin test^12^ and increased the analgesic effects of morphine in a rat model of neuropathy.^30^ Additionally, morphine inhibited the increased c‐fos expression induced by formalin administration, but naloxone preadministration antagonized the effect.^31^ Altogether, LT reduced pain‐related behavior in the second phase through increased endogenous MOR agonists release, which suppressed ERK activation and c‐fos expression in the dorsal horn.

The pERK expression in the Nal + formalin group was significantly increased compared to the formalin group and the LT + formalin group but with no significant increase in pain‐related behaviors or c‐fos expression. Acute pain has elevated β‐endorphin levels in the plasma and cerebrospinal fluid (CSF) of animals and humans.^32^, ^33^ Therefore, formalin‐induced β‐endorphin could have upregulated the pERK expression in each formalin‐treated group. The significant increase in pERK expression in the Nal + formalin group may be due to naloxone antagonizing the formalin‐induced β‐endorphin. Expressions of pERK and c‐fos did not correlate because c‐fos expression is detectable in spinal neurons 30 min after formalin injection and peaks at approximately 2 h.^24^ Thus, the unclear association of the increase in pERK with c‐fos expression could explain this observation. However, the absence of association between the pain‐related behavior duration and the change in pERK expression in the Nal + formalin group required further investigation in future studies.

Conversely, the increase of phase 2 pain‐related behaviors, and both HT‐induced pERK and c‐fos, was shown to be antagonized by naltrindole, but not by naloxone. Similar to our results, Sluka et al. revealed that naltrindole antagonized the analgesic effects of HT on acute inflammatory pain.^7^, ^9^ Additionally, DOR activation in the formalin‐induced acute pain model significantly reduced substance P release through a direct effect on primary neurons and attenuated pain.^34^ Further, a DOR agonist inhibited the increase in formalin‐induced c‐fos expression, but naltrindole preadministration antagonized this effect.^34^ These indicate that the inhibitory effects on ERK activation and c‐fos expression by HT may include increased release of endogenous DOR agonists.

TENS suppressed pain‐related behavior in the second phase (Experiment 1), thus the period from the end of TENS treatment to the end of the second phase can be considered as the duration of the preemptive analgesic effect of TENS. LT promoted significant preemptive analgesic effects for 90 min after TENS, but the effects diminished within 120 min in Experiment 2. Previous studies revealed that the half‐life of healthy human β‐endorphin was 37 min in plasma and approximately 95 min in CSF.^35^ The half‐life of β‐endorphin in sera was 6.2 ± 1.6 h in normal rats,^36^ indicating that the analgesic effects of LT do not disappear immediately after TENS treatment.^36^ Sluka et al. revealed that the analgesic effect of LT on inflamed animals lasted for 4 h.^7^ Evidence has indicated that once β‐endorphin levels in rat CSF increased, it persisted for up to 18 h.^37^ The duration of the effects of LT treatment should be dependent on the half‐life of β‐endorphins released after LT, considering the consistent duration of the preemptive analgesic effects of LT in our experiment with the half‐life of β‐endorphins reported previously.

Conversely, the preemptive analgesic effects of HT lasted for at least 120 min. However, Sulka revealed that the analgesic effect of DOR‐mediated HT lasted for 4 h in acute inflammatory pain model rats.^7^ Further, preemptive analgesic effects of HT in humans persisted up to 4 h postoperatively.^5^ These reports indicated that HT may have a longer‐lasting effect although the current study only investigated the analgesic effects of HT up to 120 min.

A limitation of this study is a lack of investigation of the involvement of inflammatory factors in the spinal dorsal horn in the TENS effects. Previous studies revealed an increase in inflammatory cytokines (tumor necrosis factor‐α and interleukins 1β and 6) in the spinal cord within 60 min after formalin administration,^38^, ^39^ with no morphological changes in glial cells.^38^, ^40^ Furthermore, Jang et al. revealed an increase in activated NFkB and IKB, and an increase in pERK and c‐fos expressions 40 min after formalin administration in the spinal dorsal horn.^38^ These reports indicate that TENS may suppress the expression and secretion of inflammatory factors. Therefore, future studies need to investigate the activation and expression of inflammatory factors in the spinal dorsal horn in this model.

## 5 CONCLUSION

LT and HT demonstrated preemptive analgesic effects for acute inflammatory pain by inhibiting ERK phosphorylation and c‐fos expression in the dorsal horn of the spinal cord, with MOR and DOR mechanisms significantly contributing to the analgesic effects of LT and HT, respectively. These effects lasted for at least 90 min for LT and 120 min for HT. Consequently, LT and HT may provide effective options for preventing postoperative pain in surgeries of relatively short duration.

## CONFLICT OF INTEREST STATEMENT

The authors declare no conflicts of interest.
